# The Effects of Neuromonitoring and Cerebrolysin Administration on Outcomes in Patients with Traumatic Brain Injury—An Interventional Pilot Study

**DOI:** 10.3390/jcm13020353

**Published:** 2024-01-08

**Authors:** Konrad Jarosz, Klaudyna Kojder, Karolina Skonieczna-Żydecka, Agata Andrzejewska, Joanna Sołek-Pastuszka, Anna Jurczak

**Affiliations:** 1 Anesthesiology and Intensive Care, University Hospital no. 1 Unii Lubelskiej, 71-252 Szczecin, Poland; jaroszki@interia.pl (K.J.); klaudynakojder@gmail.com (K.K.); joanna.solek.pastuszka@pum.edu.pl (J.S.-P.); 2 Department of Biochemical Sciences, Pomeranian Medical University, 71-460 Szczecin, Poland; 3 Department of Specialist Nursery, Pomeranian Medical University, Zolnierska 48, 71-210 Szczecin, Poland; anna.jurczak@pum.edu.pl

**Keywords:** traumatic brain injury (TBI), Cerebrolysin, Amantadine, neuromonitoring, near-infrared spectroscopy (NIRS), optic nerve sheath diameter (ONSD), intracranial pressure (ICP)

## Abstract

Introduction: Traumatic brain injury (TBI) is one of the most common causes of death and an important burden to the worldwide healthcare system and society. There is a lack of guidelines for types of monitoring or neuroprotective therapy. The aim of this pilot study was to assess its feasibility and, furthermore, to evaluate the impact of Cerebrolysin on the following clinical outcomes: length of stay, Glasgow Outcome Scale (GOS) and mortality. Methods: A cohort of 56 patients was included in this non-randomised, real-time, pre–post-interventional study. The patients were assessed with the Glasgow Coma Scale (GCS) and divided into two groups: severe (GCS < 8) and non-severe (GCS > 8). After the radiological examination (CT scan), the patients were qualified for an immediate neurosurgical procedure if needed. The patients were admitted to the intensive care unit, where a standardised protocol for TBI treatment was implemented. Additional neuromonitoring was applied. Results: There were 56 patients (19 females; 33.9%), of which 41 were considered severe cases; the patients were allocated to the Cerebrolysin (*n* = 25) or control groups (*n* = 31). In a generalised linear model (GLM) approach, the use of Cerebrolysin was associated with a decrease in the probability of death in non-severe patients (by 0.333 (standard error (SE) = 0.157, *p* = 0.034)) but not in severe patients (estimate (Est.) = −0.115, SE = 0.127, *p* = 0.364). Patients who received Cerebrolysin and who were neuromonitored had favourable outcomes and better survival rates. Conclusions: A multimodal treatment approach with monitoring and Cerebrolysin may have a beneficial effect on patients with less severe TBIs; however, the present study has multiple limitations, and further research is needed.

## 1. Introduction

Traumatic brain injury (TBI) is a heterogeneous disease. The mechanism of injury, pathophysiology and location can be radically different in different cases [1]. Across the world, the main causes of TBIs vary by population [2,3], so the approaches to treatment also vary, including their monitoring [1,4]. Patients with TBIs who are in the low Human Development Index (HDI) are more often young and tend to suffer from skull fractures due to assault; on the other hand, patients who are in the medium and high HDI most often have TBIs caused by traffic accidents. The population of patients with TBIs in countries with very high HDI tends to be older, and the cause of trauma is often a fall. The quality of care also changes with the level of HDI. It is generally less favourable in low HDI countries due to a lack of trained personnel, monitoring or equipment [5].

TBI is associated with one of the highest rates of death and failure to re-integrate into society [6]. The need for rapid medical intervention and rehabilitation in the treatment of patients with TBIs is particularly emphasised in the latest reports in the medical literature [7]. One of the milestones in the treatment of TBIs was the introduction of the Brain Trauma Foundation (BTF) guidelines [8]. The first BTF guidelines were published in 1995. With new research, evidence and trials available, the guidelines were updated and covered more areas of interest. The BTF guidelines’ implementation significantly reduced the mortality rate [9]. When medical personnel implement the BTF guidelines, mortality falls as much as 50%. However, post-TBI mortality still remains high. New consensuses are needed for further improvements, like the recent report from the Seattle Conference in 2020 [10]. Most algorithms are concerned with standards of neurological and intensive care and surgical treatment [10]. However, there is no consensus on neuroprotective treatment and types of neuromonitoring.

Cerebrolysin is a low-molecular-weight neuropeptide and free amino acid solution obtained from purified porcine brain proteins. Its beneficial influence on the outcome of patients diagnosed with TBIs has been proven [11]. It has been shown to have neuroprotective properties in both in vitro and in vivo conditions. From the molecular point of view, Cerebrolysin influences glutamatergic, GABAergic and cholinergic transmission by acting on different targets. It modulates and directs the nervous anti-inflammatory response by mimicking the activity of neurotrophic factors [11]. In animal studies, Cerebrolysin shows its long-lasting anti-inflammatory efficacy by reducing astrogliosis and stimulating neurogenesis [12]. In TBI models, introducing Cerebrolysin improves blood–brain barrier integrity by enhancing tight junction protein levels and decreases pro-inflammatory cytokines, which was also demonstrated by in vitro studies [13,14].

Amantadine is a synthetic, tricyclic amine of the adamantanes. Amantadine multi-directionally participates in the intensification of dopaminergic transmission. It affects the synthesis, turnover, uptake and synaptic release in animal studies and synthesis in human studies. It plays a role in the modification of both pre- and post-synaptic transmission. Amantadine is a low-affinity, non-competitive antagonist of NMDA receptors, which evokes effects on glutamatergic transmission. The effects on acetylcholine, serotonin, norepinephrine and phosphodiesterase activity, which are involved in modulating inflammation, remain unclear [15,16]. Since its origin, it has had different roles in the treatment of influenza, Parkinson’s disease and many other pathologies, especially with movement disorders [17]. Recently, it has also been researched, according to a few reports, on its potentially beneficial role during COVID-19 infection [18]. Several trials, including meta-analysis results, showed its beneficial use in patients with TBI diagnoses [19].

The gold standard for neuromonitoring is—apart from clinical neurological examination—the intracranial pressure (ICP) measurement. According to the BTF and Seattle Guidelines, it is advised to target the treatment for a specific ICP value and cerebral perfusion pressure (CPP) [8,10]. Without these measurements, clinicians can only observe and target the treatment on the basis of mean arterial pressure (MAP). In recent years, more data concerning non-invasive methods were published, but most of them are not yet included in the official guidelines due to a lack of randomised studies. Non-invasive methods of monitoring provide additional data about patients’ conditions, and, what is more, they often correspond with the value of ICP [20]. Still, we need more data to prove its reliability and accuracy.

The gold standard for the neuromonitoring of patients after TBIs is the direct measurement of the ICP. It is recommended both by the Brain Trauma Foundation guidelines and Seattle Consensus, which provide clinicians with algorithms, including special treatment tiers and targets [7,10]. ICP is an invasive method in which a catheter is placed in the brain parenchyma or in the lateral ventricles. In the latest reports, ventricle catheters are associated with a higher rate of infection; however, it does not affect the mortality rate, and the general outcome remains the same as with the parenchymal catheters [21]. Based on the measured ICP values, the CPP can be determined, and it should be kept between 60 and 70(80) mmHg, especially in patients with impaired autoregulation [10]. The most common complications are bleeding, infections and incorrect placement of the catheter, which may cause misreading [21].

In the current literature, there is not much information about the influence of Cerebrolysin, other neuroprotective pharmacotherapy or neuromonitoring on TBI patients.

The aim of this study was to assess the impact of Cerebrolysin and/or Amantadine administration and neuromonitoring on the final neurological outcome, LOS and mortality in patients with TBI. 

## 2. Materials and Methods

### 2.1. Design of the Study and Aim

This study was conducted as a non-randomised, real-time, pre-post interventional one of pilot nature. This study was registered in Clinical Trial database: NCT05807503. The primary outcome was this study’s feasibility, and the secondary outcome was the analysis of possible clinical implications of Cerebrolysin usage.

### 2.2. Participants

This study was conducted with patients diagnosed with TBI, defined as a closed injury, i.e., a disruption of the normal function of the brain caused by an external force [6]. All patients aged over 18 years who were diagnosed with TBI and admitted to the University Hospital no. 1 in Szczecin, Poland, between 2021 and 2022 were included in this study.

The following exclusion criteria were applied:

- Age < 18 years;
- Proven allergy to Cerebrolysin;
- Acute renal failure;
- Pregnancy;
- Multi-organ trauma;
- Death within 48 h of admission.

### 2.3. Clinical Management

TBI patients were clinically assessed in the emergency department and, after the radiological examination (CT scan), were qualified for immediate neurosurgical intervention if necessary. They were then admitted to the intensive care unit, where a standardised protocol for TBI management was implemented [8,10].

Patients were initially assessed using the Glasgow Coma Scale (GCS) [22]. In the present study, we divided the patients into two groups: severe (GCS < 8) and non-severe (GCS > 8).

In the intensive care unit (ICU), most of the patients were sedated with propofol and fentanyl as a continuous infusion. Routine neurological examination was performed several times a day to assess the neurological reflexes. Additional neuromonitoring was performed in the form of ICP, NIRS, EEG, SjO2 or ONDS as required. In addition, interventional treatment was initiated at this stage, as detailed in Section 2.4. Intravenous osmotic therapy was administered under the control of osmolality, either calculated or measured by laboratory tests. Patients were initially ventilated invasively with the intubation tube according to recommendations for lung-protective ventilation. Positive end-expiratory pressure (PEEP) did not exceed 8 mmHg in all cases. Patients with altered consciousness or failed extubation attempts underwent tracheostomy.

During the first period of the acute post-trauma phase, MAP was maintained above 85 mmHg in patients without ICP monitoring. Vasopressors were used to maintain blood pressure at the predefined target: norepinephrine and/or vasopressin analogues. Patients with ICP monitoring were treated with vasopressors to maintain CPP within the recommended range—above 60 mmHg.

**Figure 1:**
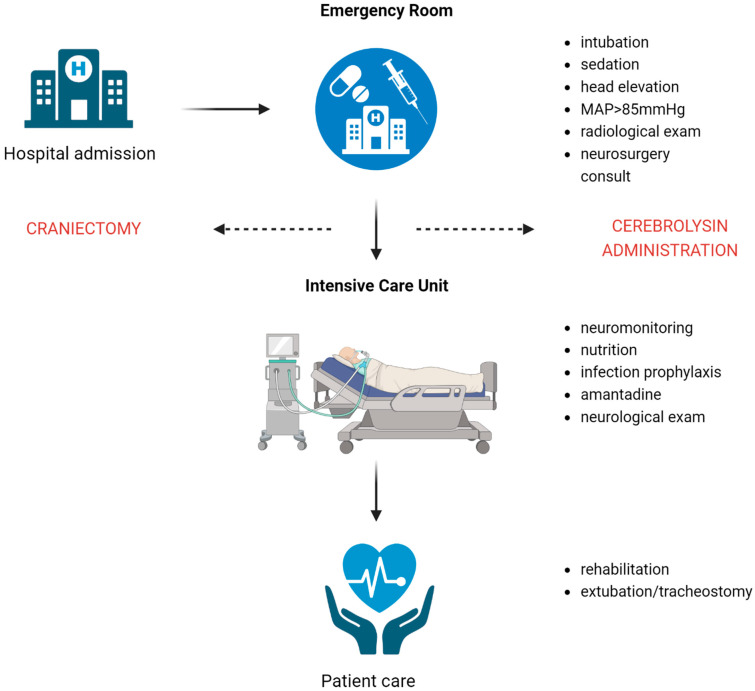
Treatment scheme of a TBI patient.

Enteral nutrition was started via a nasogastric tube the day after admission. Patients with severely altered consciousness or with persistent dysphagia at the end of the treatment underwent a percutaneous gastrostomy performed via a minimally invasive endoscopic approach. Figure 1 illustrates the treatment scheme of a TBI patient in the present study.

### 2.4. Intervention

The internal standardised operating procedure for starting Cerebrolysin treatment was used. Cerebrolysin was started as soon as possible (but no later than 24 h after the injury) at a daily dose of 30 mL or 50 mL intravenously and continued for the duration of the ICU stay at the discretion of the intensive care physician. 

Amantadine is a part of the treatment for TBI patients. We were not able to collect a properly powered group of patients receiving this drug, but we decided to see if there was a possible association and how it affected the outcomes.

### 2.5. Outcomes

Clinical outcomes included Glasgow Outcome Scale (GOS) [23], length of stay (LOS) and mortality.

### 2.6. Statistical Analysis

Two-sided *p* < 0.05 indicates a statistically significant difference. Continuous variables that followed a normal distribution were expressed as mean ± standard deviation (SD). Continuous variables that were not normally distributed were defined as median and interquartile range (IQR). Categorical data were expressed as numbers (percentages) and compared using chi-square or Fisher’s exact test. Statistical analyses were performed using Med Calc statistical software version 20.210 (Ostend, Belgium).

In a multi-variable analysis, we used a generalised linear model with different families of distributions. Specifically, we used a Gaussian family for the quantitative dependent variable (LOS), a Poisson family for ordinal variables (GOS) and a binomial family for dichotomous variables (death).

We conducted this analysis both with and without interaction terms, including TBI severity and other treatment factors. To help understand the results of the models, we calculated marginal effects using the “marginal effects” package version 0.15.1 in R. Effect sizes: Cohen’s d, Glass’s delta and Hedges’ g were calculated. All of these analyses were performed using the R programming language (R Core Team, 2022), which is a statistical computing environment (https://www.R-project.org/).

## 3. Results

### 3.1. Patient Characteristics

Data from 56 patients (19 females; 33.9%) allocated to the Cerebrolysin group (*n* = 25) or control group (*n* = 31) were included. There were 41 severe cases (74.5%) based on GCS score, but one patient’s GCS was not assessed on admission. The mean age in the whole group was 59.07 ± 16.78 years (median: 62.0; IQR: 44.5–70.0). There were no age or gender differences in the GCS subgroups (*p* = 0.75 and *p* = 0.59, respectively). Amantadine was administered in 10 patients (17.9%). Neurosurgical craniotomy was performed in 53 patients (94.6%), and additional neuromonitoring was performed in 18 patients (32%). As shown in Table 1, no significant differences in TBI severity were observed with these treatments.

### 3.2. Cerebrolysin Efficacy

**Figure 2:**
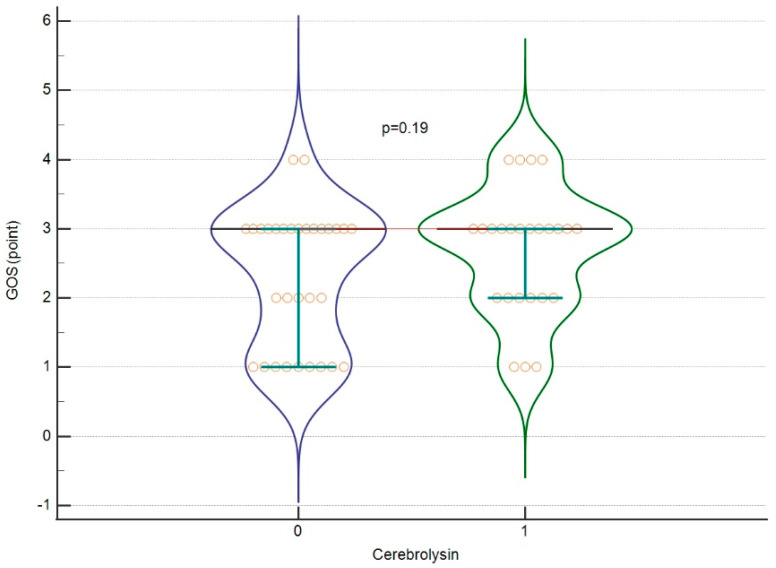
GOS in relation to Cerebrolysin treatment. A violin plot showing medians and IQR. Orange circles represent individual cases. The red horizontal line connects the medians.

**Figure 3:**
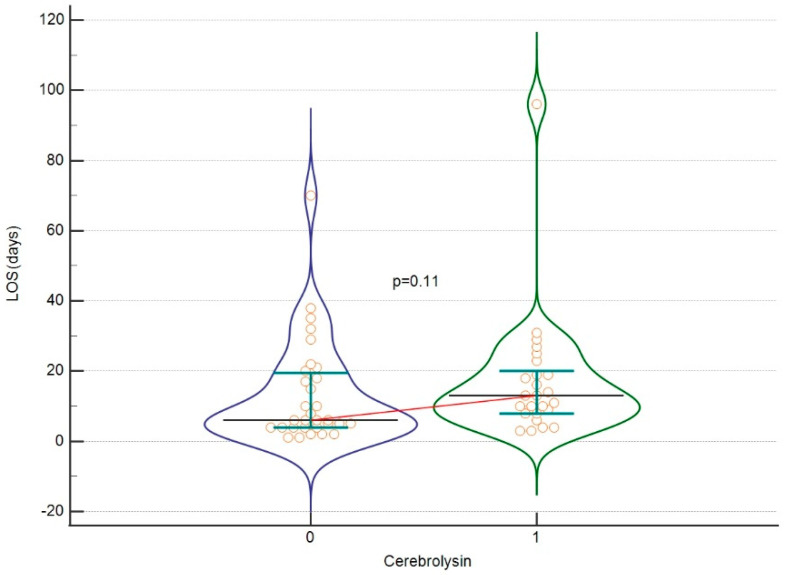
LOS in relation to Cerebrolysin treatment. A violin plot showing medians and IQRs. The orange circles represent individual cases. The red horizontal line connects the medians.

The mean GOS score for all severe and non-severe patients was 2.48 ± 0.95 points, while the LOS was 15.02 ± 16.52 days. In the first set of analyses, we assessed whether Cerebrolysin affected the raw GOS score in all patients without dividing them into severe and non-severe groups. The effect sizes of Cerebrolysin treatment according to the severity of TBI are included in Table 2. No significant differences were found (*p* = 0.197). Similar results were obtained for LOS (*p* = 0.11; Figure 2 and Figure 3). No significant differences were found between severe and non-severe patients for either measure. The effect sizes for these calculations are provided in Table 3.

We also used generalised linear models (GLMs) to assess the effect of Cerebrolysin on the outcomes (LOS, GOS, death) while controlling for other variables (e.g., age, gender, severity of injury). Using the GLM framework also allowed us to explore possible interactions between Cerebrolysin, the severity of injury and other treatment modalities for TBI. Table 3 and Table 4 show the details.

We found no significant effect of Cerebrolysin on GOS or LOS, neither without nor with interaction with TBI severity, use of Amantadine, craniectomy or neuromonitoring.

In the final set of data analysis, using Chi2 tests, we found that Cerebrolysin did not affect mortality in either severe or non-severe patients. The data are shown in Table 5.

Using GLM, we found that the effect of Cerebrolysin on survival may vary according to the TBI severity, the use of Amantadine and neuromonitoring. The use of Cerebrolysin was associated with a decrease in the probability of death in non-severe patients (by 0.333 (standard error (SE) = 0.157, *p* = 0.034) but not in severe patients (estimate (Est.]) = −0.115, SE = 0.127, *p* = 0.364). Similarly, the use of Cerebrolysin was beneficial in terms of survival in patients not taking Amantadine (Est. = −0.228, SE = 0.115, *p* = 0.047) and undergoing neuromonitoring (Est. = −0.600, SE = 0.280, *p* = 0.032). Details are shown in Table 6.

## 4. Discussion

TBI is a heterogeneous condition. From the moment of injury to the patient’s recovery, each element of treatment has a significant impact on the final outcome. Errors, as well as correct decisions at each stage, can affect the clinical outcome. In pre-hospital care, quick decisions are of paramount importance, including airway protection and proper ventilation, avoidance of hypotension and the prevention of cerebral oedema. Proper triage and rapid transport to the nearest facility with neurotrauma expertise are critical. From the outset, the most important aspects of hospital care are rapid radiological assessment and decisions about possible surgical intervention. During the stay in the ICU, the basics of treatment, such as sedation, ventilation and maintaining proper haemodynamic stability, are crucial for the patient’s survival.

As a result, there is a growing body of literature demonstrating the impact of multimodal protocols on the treatment and diagnosis of TBI patients [24,25]. Previous standards of care using isolated treatment modalities are increasingly being replaced by multimodal approaches [20].

The multimodal approach to TBI treatment includes not only the basic therapy but also an attempt to use all available modalities [26]. The monitoring of several different parameters and trends helps to achieve a more personalised therapy, which increases the safety and efficacy of treatment. When talking about multimodal treatment, it is worth mentioning pharmacotherapy with different molecular targets. Of no less importance is intensive rehabilitation, not only in the post-acute phase of TBI but starting while the patient is still in the ICU. By using as many methods as possible to improve patient care, this study seems to show that it is possible to affect a variety of treatment targets. A multimodal approach may produce better results not only in terms of mortality but also in terms of cognitive outcome [27].

Our study showed that neuromonitoring combined with Cerebrolysin may have a beneficial effect on mortality in less severely affected patients with TBI. Due to the pilot nature of our study, the results we achieved need to be re-evaluated and validated in studies with larger numbers of participants.

In the context of neuroprotective treatment, there is no published gold standard in the available literature. Cerebrolysin is a drug with a proven positive effect on the outcomes (GOS, mRankin scale) in patients with TBI. The CAPTAIN II and CAPTAIN meta-analyses confirmed a positive effect of Cerebrolysin on global outcome, cognition, attention and depression symptoms [28,29]. The results of our study show that the use of Cerebrolysin was associated with a reduction in the likelihood of death in non-severe patients (classified by the initial GCS score). We also found that the use of Cerebrolysin was beneficial in terms of survival in patients not taking Amantadine and undergoing neuromonitoring. At the cellular level, Cerebrolysin increases the number of neuroblasts, leads to the promotion of neurogenesis in the dentate gyrus and also reduces astrogliosis in the corpus callosum, cortex, dentate gyrus, and CA1 and CA3 regions [13]. In updated guidelines, Cerebrolysin has been recommended for use in early motor rehabilitation after acute ischemic stroke by the European Academy of Neurology and the European Federation of Neurorehabilitation Societies [30]. It has also recently been identified as an agent that could be used to achieve better cognitive outcomes in patients after acute brain injury (ABI) in an evidence-based review of research data up to 2020 [31]. The observation about Amantadine is surprising. Amantadine has a positive effect on cognition in TBI patients in the medium term [16]. We can also find the first reports of a beneficial additive effect of Amantadine and Cerebrolysin used together in patients with acquired brain injury [32]. The synergistic effect of two different pharmacological agents could be explained by the fact that they act on different receptor targets. Cerebrolysin acts as an agent that mimics the activity of neurotrophic factors such as NTF, and Amantadine acts on the NMDA and dopamine pathways. These two modes of action are a part of the pathological mechanisms of TBI and can be modulated in the early and late stages of recovery. Cerebrolysine and Amantadine have been shown to have a beneficial effect on the cognitive outcome of TBI, even in the post-acute phase of brain trauma [33,34]. Lee et al. reported a retrospective case–control study describing 84 TBI patients who were treated in two groups: Amantadine only and Cerebrolysin plus Amantadine. After 4 weeks of follow-up, the combined treatment group showed a greater increase in the Coma Recovery Scale-Revised (CRS-R) score [32].

Currently, ICP measurement is the gold standard for monitoring patients after TBI. SjO2 monitoring is also mentioned in the BTF guidelines [8,10]. However, due to the invasiveness of these procedures and possible complications, attention is also being paid to non-invasive methods. To date, there is a lack of conclusive data and recommendations, so non-invasive methods are not part of a standardised and recommended protocol for monitoring TBI patients. However, they may correspond to increased ICP, and some of these methods anticipate deterioration of the clinical condition of patients. It is worth considering the use of several of these methods to monitor the patient more closely and obtain more information about the patient [35].

The main limitation of our study is the small sample size. However, given the pilot nature of our clinical trial, which is, in fact, exploring uncharted research territory, it was sufficient to detect significant differences and provide preliminary evidence of the efficacy of a Cerebrolysin-based approach to treating TBI patients. Another important limitation is the non-randomised, single-centre nature of this study. Our cohort of patients was heterogeneous in terms of TBI severity, which also implied different monitoring and surgical treatments. As a result, there was also selection bias due to different treatment modalities.

## 5. Conclusions

Cerebrolysin and multimodal neuromonitoring may have a beneficial effect on patients with non-severe TBI, but due to many limitations of our study, we cannot make any recommendations, and for now, it should preferably be used in clinical trials.

## Figures and Tables

**Table 1:** Treatment characteristics of TBI patients (in one patient, GCS on admission was not assessed).

	Amantadine		
GCS_qualitatively	NO	YES	*p*
Non-severe	11	3	0.55
Severe	35	6	
	**Cerebrolysin**		
GCS_qualitatively	NO	YES	*p*
Non-severe	9	5	0.49
Severe	22	19	
	**Craniectomy**		
GCS_qualitatively	NO	YES	*p*
Non-severe	0	14	0.40
Severe	2	39	
	**Monitoring**		
GCS_qualitatively	NO	YES	*p*
Non-severe	10	4	0.82
Severe	28	13	

**Table 2:** Effect sizes of Cerebrolysin treatment according to the severity of TBI.

	GOS			LOS		
	Cohen’s d	Glass’s delta	Hedges’ g	Cohen’s d	Glass’s delta	Hedges’ g
Severe	0.282542	0.276596	0.282071	0.182936	0.204834	0.184376
Not severe	0.887709	0.783784	0.878209	0.696779	0.501119	0.60852

**Table 3:** Cerebrolysin contrasts (yes–no) based on generalised linear model (Poisson family) for different sets of predictors with GOS as the dependent variable.

Predictor Space		Contrast Estimate	SE	*p*
Cerebrolysin ^1^ (YES vs. NO)		0.407	0.511	0.425
Cerebrolysin (YES vs. NO) ^2^	Severe	0.261	0.491	0.595
	Non-severe	0.867	0.948	0.361
Cerebrolysin (YES vs. NO) ^3^	Amantadine (Yes)	0.167	1.034	0.872
	Amantadine (No)	0.388	0.476	0.415
Cerebrolysin (YES vs. NO) ^4^	Craniectomy (Yes)	0.396	0.44	0.369
	Craniectomy (No)	−0.500	2.06	0.80
Cerebrolysin (YES vs. NO) ^5^	Neuromonitoring (Yes)	1.133	0.862	0.188
	Neuromonitoring (No)	0.107	0.579	0.853

1—age, gender, Cerebrolysin, Amantadine, craniectomy, neuromonitoring, TBI severity (no interaction); 2—Cerebrolysin, TBI severity (interaction); 3—Cerebrolysin, Amantadine (interaction); 4—Cerebrolysin, craniectomy (interaction); 5—Cerebrolysin, neuromonitoring (interaction).

**Table 4:** Cerebrolysin contrasts (yes–no) based on generalised linear model (Gaussian family) for different sets of predictors with GOS as the dependent variable.

Predictor Space		Contrast Estimate	SE	*p*
Cerebrolysin ^1^ (YES vs. NO)		6.48	5.37	0.228
Cerebrolysin (YES vs. NO) ^2^	Severe	3.39	5.08	0.504
	Non-severe	2.76	9.05	0.761
Cerebrolysin (YES vs. NO) ^3^	Amantadine (Yes)	0.167	0.621	0.788
	Amantadine (No)	0.388	0.288	0.178
Cerebrolysin (YES vs. NO) ^4^	Craniectomy (Yes)	3.48	4.66	0.456
	Craniectomy (No)	13.50	20.60	0.512
Cerebrolysin (YES vs. NO) ^5^	Neuromonitoring (Yes)	10.80	10.49	0.303
	Neuromonitoring (No)	6.41	6.11	0.290

1—age, gender, Cerebrolysin, Amantadine, craniectomy, neuromonitoring, TBI severity (no interaction); 2—Cerebrolysin, TBI severity (interaction); 3—Cerebrolysin, Amantadine (interaction), 4—Cerebrolysin, craniectomy (interaction); 5—Cerebrolysin, Neuromonitoring (interaction).

**Table 5:** The mortality of TBI patients according to the severity of injury.

Variable	Whole Group			Severe			Not Severe		
Cerebrolysin	NO	YES	*p*	NO	YES	*p*	NO	YES	*p*
ALIVE	22	21	0.12	16	16	0.38 (0.65)	6	5	0.16)
DEAD	9	3		6	3		3	0	
Amantadine									
ALIVE	35	8	0.33	27	5	0.73 (0.73)	8	3	0.32
DEAD	11	1		8	1		3	0	
Craniectomy									
ALIVE	2	41	0.36	2	30	0.44 (0.65)	0	11	n.e.
DEAD	0	12		0	9		0	3	
Neuromonitoring									
ALIVE	29	14	0.55	21	11	0.49 (0.65)	8	3	0.84
DEAD	9	3		7	2		2	1	

n.e.—not estimable.

**Table 6:** Cerebrolysin contrasts (yes–no) based on generalised linear model (binomial family) for different sets of predictors with death as dependent variable.

Predictor Space		Contrast Estimate	SE	*p*
Cerebrolysin ^1^ (YES vs. NO)		−0.199	0.13	0.126
Cerebrolysin (YES vs. NO) ^2^	Severe	−0.115	0.127	0.364
	Non-severe	−0.333	0.157	0.034
Cerebrolysin (YES vs. NO) ^3^	Amantadine (Yes)	0.167	0.152	0.273
	Amantadine (No)	−0.228	0.115	0.047
Cerebrolysin (YES vs. NO) ^4^	Craniectomy (Yes)	−1.700	0.109659	0.122
	Craniectomy (No)	−1.59 × 10^−16^	0.000114	1.000
Cerebrolysin (YES vs. NO) ^5^	Neuromonitoring (Yes)	−0.600	0.280	0.032
	Neuromonitoring (No)	−0.050	0.151	0.740

1—age, gender, Cerebrolysin, Amantadine, craniectomy, neuromonitoring, TBI severity (no interaction); 2—Cerebrolysin, TBI severity (interaction); 3—Cerebrolysin, Amantadine (interaction); 4—Cerebrolysin, craniectomy (interaction); 5—Cerebrolysin, neuromonitoring (interaction).

## Data Availability

All the data are available to be shared after directly contacting the corresponding author.
